# Lung Secretoglobin Scgb1a1 Influences Alveolar Macrophage-Mediated Inflammation and Immunity

**DOI:** 10.3389/fimmu.2020.584310

**Published:** 2020-10-01

**Authors:** Min Xu, Wei Yang, Xuanchuan Wang, Deepak Kumar Nayak

**Affiliations:** ^1^Department of Surgery, Washington University School of Medicine, St. Louis, MO, United States; ^2^Department of Genetics, Washington University School of Medicine, St. Louis, MO, United States; ^3^Department of Urology, Zhongshan Hospital, Fudan University, Shanghai, China; ^4^Interdisciplinary Oncology, University of Arizona College of Medicine, Phoenix, AZ, United States

**Keywords:** club cell, alveolar macrophage, lung surfactant, SCGB1A1, Clara cell secretory protein, cytokine storm

## Abstract

Alveolar macrophage (AM) is a mononuclear phagocyte key to the defense against respiratory infections. To understand AM’s role in airway disease development, we examined the influence of Secretoglobin family 1a member 1 (SCGB1A1), a pulmonary surfactant protein, on AM development and function. In a murine model, high-throughput RNA-sequencing and gene expression analyses were performed on purified AMs isolated from mice lacking in *Scgb1a1* gene and were compared with that from mice expressing the wild type *Scgb1a1* at weaning (4 week), puberty (8 week), early adult (12 week), and middle age (40 week). AMs from early adult mice under *Scgb1a1* sufficiency demonstrated a total of 37 up-regulated biological pathways compared to that at weaning, from which 30 were directly involved with antigen presentation, anti-viral immunity and inflammation. Importantly, these pathways under *Scgb1a1* deficiency were significantly down-regulated compared to that in the age-matched *Scgb1a1-*sufficient counterparts. Furthermore, AMs from *Scgb1a1*-deficient mice showed an early activation of inflammatory pathways compared with that from *Scgb1a1*-sufficient mice. Our *in vitro* experiments with AM culture established that exogenous supplementation of SCGB1a1 protein significantly reduced AM responses to microbial stimuli where SCGB1a1 was effective in blunting the release of cytokines and chemokines (including IL-1b, IL-6, IL-8, MIP-1a, TNF-a, and MCP-1). Taken together, these findings suggest an important role for *Scgb1a1* in shaping the AM-mediated inflammation and immune responses, and in mitigating cytokine surges in the lungs.

## Introduction

The ongoing pandemic of Coronavirus disease 2019 (COVID-19) caused by severe acute respiratory syndrome coronavirus 2 (SARS-CoV-2) has already affected over 30 million people across the globe resulting in more than one million deaths (1, 2). It produces a significant amount of severe illnesses that overwhelm health care infrastructure. Early studies have shown that the prognosis of COVID-19 may be variable among different populations and age groups. Particularly, it disproportionately affects the elderly and patients with preexisting conditions including chronic obstructive pulmonary disease (COPD) and hypertension compared to any other conditions (3, 4). Mechanisms of SARS-CoV-2 persistence and pathogenicity remains largely unknown; however, its widespread infection and associated fatalities outnumber the past coronavirus outbreaks such as the Middle East respiratory syndrome-related coronavirus and severe acute respiratory syndrome-associated coronavirus. The rapid decline in lung function and some of the COVID-19 patients requiring oxygen support and/or mechanical ventilation has been attributed to cytokine storm (5). There is no targeted anti-viral therapy currently available for COVID-19 and efforts to develop vaccines are ongoing. In this context, examining the cellular, and/or molecular interactions within the lung microenvironment may provide useful insights into the pathogenesis and possible therapeutics of COVID-19 and other infectious respiratory diseases.

Alveolar macrophages (AMs) are stationary cells of embryonic origin that constitute greater than 95% of the phagocyte pool in the alveolar space (6, 7). Consequently, AMs perform crucial immune surveillance at the respiratory surface providing the first line defense against airborne pathogens, pollutants (8–12), and clearing of cellular debris (13). AMs function as professional antigen presenting cells in eliciting antigen specific T cells and antibodies targeting pathogens (14, 15) and autoantigens (16, 17). AMs also can respond to various microbial stimuli producing an array of cytokines and chemokines that may influence the landscape of inflammation and immunologic outcomes (16–18).

On the other hand, club cells are a significant contributor to the homeostatic and reparative processes in the lungs (19). These non-ciliated and non-mucous-producing cells in the bronchiolar epithelium can differentiate into pulmonary epithelial and endothelial cells following tissue injury (20–23), and via secreted secretoglobin family 1A member 1 (SCGB1A1) protein, a component of the pulmonary surfactant, can exert anti-inflammatory and anti-fibrotic functions (24). Specifically, SCGB1A1 binds and sequesters key inflammatory mediators of airway diseases including prostaglandins (25, 26), phospholipase (PL) A2 (27–29) and PLC (28), and inhibits activation and translocation of NF-κB (30, 31). Lung infection by viruses and bacteria are known to elicit greater inflammatory responses in the absence of SCGB1A1 (32–34), whereas respiratory distress decreases SCGB1A1 levels following acute lung injury (35); exposures to pollutants (36), cigarette smoke (37) and ozone (38); lung allograft rejection (39–41); respiratory infections (42, 43); and chronic lung diseases (44, 45). Overexpression of *Scgb1a1* in airways has shown to limit ventilator induced lung injury and inflammation (46), and supplementation of exogenous SCGB1A1 has mitigated the increased proinflammatory cytokines and inflammatory buildup caused by *Scgb1a1* germline-deficiency (24).

Because AMs are non-migratory cells that adhere to the alveolar epithelium (47), they are constantly immersed in pulmonary surfactant and are likely to be influenced by the surfactant components. Under steady state, AMs exhibit a non-inflammatory phenotype while SCGB1A1 occurs at its physiologic maximum. Club cells, on the other hand, succumb to respiratory distress resulting in decreased SCGB1A1 levels. As the crosstalk between club cells and AMs is largely unknown, for the first time in the present study, we examine the transcriptomic profiles of mouse AMs during development and compare the shift in gene expression under *Scgb1a1*-deficiency. Furthermore, we also analyze the effects of SCGB1A1 protein on purified AMs delineating therapeutic implications of SCGB1A1 in inflammatory and fibrotic lung diseases.

## Materials and Methods

### Experimental Animals

Wild type (WT, *Scgb1a1*^+/+^) C57BL/6 mice were procured from Charles River Laboratories and Uteroglobin gene knockout (KO) mice (*Scgb1a1*^–/–^) (48) were obtained from National Institutes of Health. Mice were housed at the Washington University School of Medicine according to institutional guidelines and approved protocols. Mice from WT and KO groups were euthanized at 4, 8, 12, and 40 weeks of age [respectively, equivalent to 6 months, 12 years, 20 years, and 40 years in human age (49)] and relevant samples were collected for further study (*n* = 3/group).

### Flow Cytometry Analysis

We performed multicolor flow cytometry to analyze expression of a panel of phenotypic and functional markers. Bronchoalveolar lavage (BAL) cells were isolated per our established protocol (50) and incubated with Fc block (BD Bioscience) to prevent non-specific antibody binding. They were incubated with fluorophore tagged antibodies for CD45, CD11c and Siglec-F, and were analyzed with a BD LSR Fortessa cell analyzer (BD Bioscience). The functional states of AM at 4, 8, 12, and 40 weeks were compared between the KO and WT groups. Single stain control and fluorescence minus one control were included in every study and data were analyzed by FlowJo v10.6.2 (BD Life Sciences).

### AM Isolation and RNA Purification

Alveolar macrophages, defined as CD45^+^, CD11c^hi^, and Siglec-F^hi^ granular cells, were isolated from BAL fluid via flow cytometry cell sorting technique following our previous study (50), and age-matched samples were collected and processed on the same day to minimize batch-to-batch variation. AMs were isolated individually from three mice per group and were sorted directly into RNA Lysis buffer using a PureLink RNA kit (Thermo Fisher Scientific). Total RNA was treated by DNase I (Thermo Fisher Scientific), quantitated by Nanodrop (Thermo Fisher Scientific), and stored at -80°C.

### RNA Sequencing and Bioinformatics Analysis

Three biologic replicates per group were included to ensure a strong statistical power for detecting the inter- and intra-group variations. Total RNA (up to 100 pg) was subjected to the picoRNA workflow (Cofactor Genomics). A poly-A library was constructed and 6 × 10^7^ single-end reads were performed. Following RIN score (>7) determination and enrichment for mRNA, RNA sequencing was performed (Cofactor Genomics) for 60 million single-end reads covering >75 base pairs/read. RNA-seq reads were then aligned to the 76 primary assemblies for *Mus musculus* with STAR version 2.5.1a. Gene counts were derived from the number of uniquely aligned unambiguous reads by Sub read: feature Count version 1.4.6-p5. All gene counts were then imported into the R/Bioconductor package EdgeR and TMM normalization size factors were calculated to adjust for samples for differences in library size. The TMM size factors and the matrix of counts were then imported into the R/Bioconductor package Limma, and analyzed for differential expression using Limma/voom and sequencing data was analyzed by Genomics Suite (Partek). The data set has been submitted to Gene Expression Omnibus (GEO) with accession no GSE148647. The data were examined for changes in transcription profile in comparison with their WT counterpart by three-dimensional PCA plot, hierarchical clustering, Venn diagram, volcano plot, and profile trellis and gene ontology enrichment. Generally applicable gene set enrichment (GAGE) method was applied for pathway analysis (51) through databases including gene ontology (GO, molecular function and biological process) and Kyoto encyclopedia of genes and genome (KEGG, metabolism and disease pathway). The Benjamini and Hochberg’s False Discovery Rate (FDR) correction was performed to determine the significance of gene expression (51).

### Influence of SCGB1a1 Protein on AM Response to Inflammatory Stimuli

The effect of SCGB1a1 protein on AM responsiveness to inflammatory stimuli were studied *in vitro*. Flow sorted AMs were plated (50) in 12-well plates at 1 × 10^5^ cells/well in triplicates and were incubated with Toll-like receptor (TLR) agonists (Invivogen) 2- heat-killed *Listeria monocytogenes* (HKLM), TLR4-Lipopolysaccharide from *Escherichia coli* K12 (LPS) and TLR5- *Salmonella typhimurium* Flagellin (FLA) in presence or absence of recombinant SCGB1a1 protein at 5 μg/mL (Creative BioMart). Culture supernatant was collected and total RNA was isolated at 72 h post-stimulation. The release of cytokines and chemokines in culture supernatant was analyzed by a Bio-Plex 200 system (Bio-Rad) using multiplex immunoassays.

## Results

### Influence of Scgb1a1 Deficiency on AM Phenotype

**FIGURE 1:**
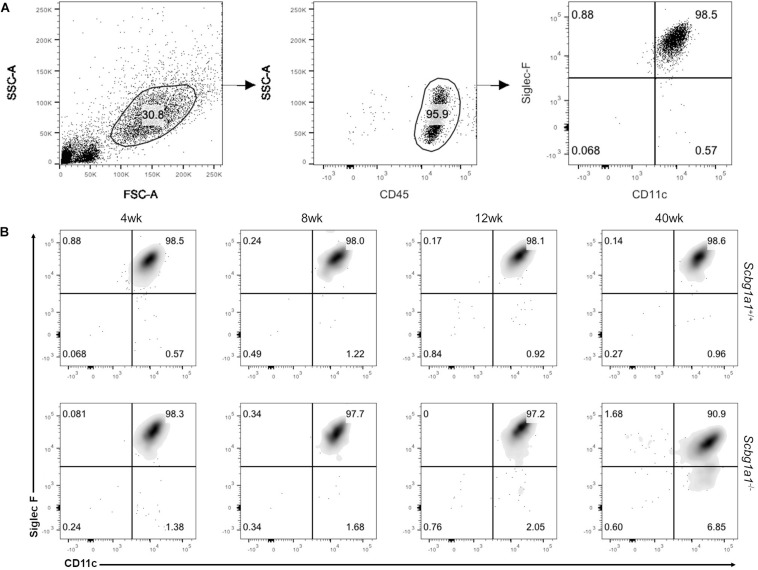
Influence of *Secretoglobin family 1A member 1* (*Scgb1a1*) deficiency on alveolar macrophage (AM) phenotypes. **(A)** Flow cytometric analysis of mouse AMs (CD45^+^, CD11c^hi^, and Siglec-F^hi^ cells) from bronchoalveolar lavage fluid. **(B)** AMs from WT (*Scgb1a1*^+/+^) and KO (*Scgb1a1*^–/^*^–^*) mice were analyzed for phenotypic differences at 4, 8, 12, and 40 weeks of age.

We evaluated the prevalence and phenotype of AMs in age, gender and strain matched *Scgb1a1*^–/–^ and *Scgb1a1*^+/+^ mice. AMs, identified as BAL cell leukocytes with expression of CD11c^hi^ and Siglec-F^hi^, were nearly identical between the KO and WT groups at earlier time points of 4 and 8 weeks (Figure 1). Interestingly, as early as 12-week, a unique population, *albeit* minor, with CD11c^hi^ and Siglec-F^*low*^ began to appear in the KO only. By 40-week, this population in KO had grown to represent ∼7% cells compared to ∼1% of that in the age-matched WT. Developmental origin and physiologic significance of the CD11c^hi^ and Siglec-F^*low*^ cells in *Scgb1a1*^–/–^ is currently unknown; however, some unrelated studies have suggested a monocytic precursor for the Siglec-F^*low*^ pulmonary macrophages (52, 53). This phenotypic difference in AMs as early as 12 weeks of age suggests a variation in myelopoiesis and/or activation status associated with *Scgb1a1* deficiency.

### Genes and Gene Sets Differentially Expressed in WT AMs

**FIGURE 2:**
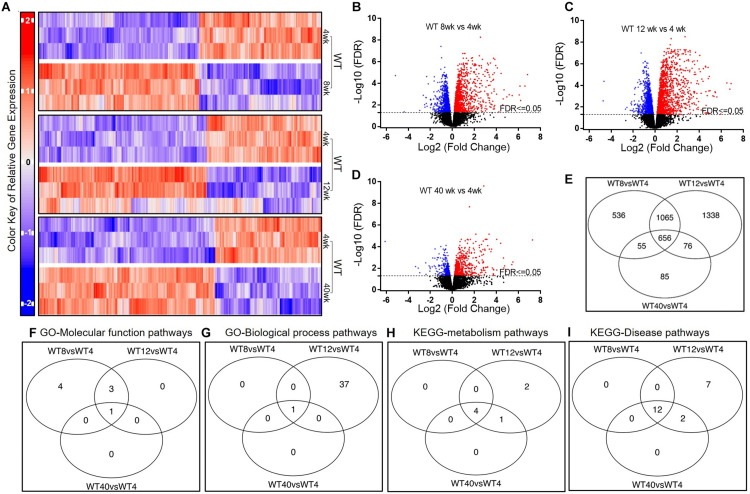
Gene expression in AMs from wild type mice across age groups. **(A)** Up-regulated and down-regulated genes in AMs at 4, 8, 12, and 40 weeks; **(B–D)** Volcano plots of significantly up-regulated (red) and down-regulated (blue) genes (FDR < 0.05); **(E)** Venn diagrams of the significantly changed genes; Venn diagrams of the significantly changed gene sets in AMs of the WT mice at different ages including GO-Molecular function pathways **(F)**, GO-Biological process pathways **(G)**, KEGG-metabolism pathways **(H)**, and KEGG-Disease pathways **(I)**. FDR < 0.05 was considered significant; FC, fold change.

To study alteration in gene expression due to aging, we compared the AM expressed genes at 8, 12, and 40 weeks with that at 4 weeks in WT mice (Figure 2A). As shown in Figures 2B–D, there were 2312 genes at 8-week, 3135 genes at 12-week, and 872 genes at 40-week significantly up-regulated compared to that at the 4-week (FDR ≤ 0.05). The overlapping pattern of gene expression was categorized in a Venn diagram indicating that 656 genes were remarkably altered at all time-points beyond 4 weeks of age (Figure 2E). Additionally, ten most significantly up-regulated and down-regulated genes along with FDR values are presented in Supplementary Table 1.

To delineate the biological significance of the shift observed in gene expression, we analyzed the significantly changed gene sets using GAGE method and screened through GO and KEGG databases (Figures 2F–I). As shown in Supplementary Table 2, there were seven and three up-regulated GO molecular function pathways at 8 and 12 weeks, respectively, in comparison to that at 4 weeks. In contrast, the ribosome related pathway was down-regulated at 8, 12, and 40 weeks compared to that at 4 weeks. Importantly, most of the GO biological processes including the innate immune response pathway were up-regulated at 12-week compared with that at 4-week. There were 37 significantly up-regulated biological processes evident at 12-week, among which 30 biological pathways were directly involved with immune defenses and inflammatory process. No significant change in GO biological process was, however, found at 40-week compared to that at 4-week. In the KEGG metabolism pathway analysis, antigen processing and presentation, phagosome, and cell adhesion molecule pathways were significantly up-regulated at 8, 12, and 40 weeks than that at 4 weeks. In the KEGG disease pathway analysis, several infectious diseases and immune/metabolism related pathways including that of Influenza A, Epstein-Barr virus (EBV), Measles and *Staphylococcus aureus*, and non-alcoholic liver disease were significantly changed at 8, 12, and 40 weeks than that at 4 weeks.

In summary, most of the pathways for immunologic and inflammatory responses were activated by 12 weeks of age. This suggests that early adult WT mice may have a more pronounced immunological defense compared to any other age groups studied.

### Significant Changes in Gene Expression in KO AMs

**FIGURE 3:**
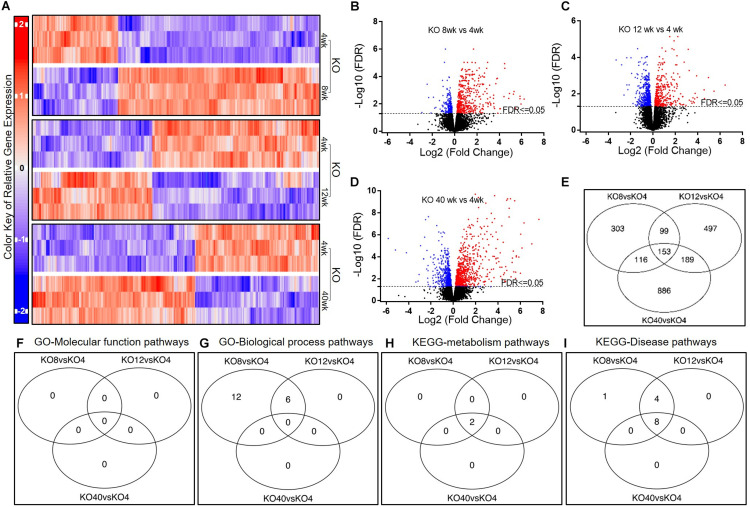
Gene expression in AMs from *Scgb1a1* KO mice across age groups. **(A)** Up-regulated and down-regulated genes in AMs at 4, 8, 12, and 40 weeks; **(B–D)** Volcano plots of significantly up-regulated (red) and down-regulated (blue) genes (FDR < 0.05); **(E)** Venn diagrams of the significantly changed genes. Venn diagrams of the significantly changed gene sets in AMs from KO mice at different ages including GO-Molecular function pathways **(F)**, GO-Biological process pathways **(G)**, KEGG-metabolism pathways **(H)**, and KEGG-Disease pathways **(I)**. FDR < 0.05 was considered significant; FC, fold change.

To assess the patterns of AM gene expression due to aging under *Scgb1a1* deficiency, we studied flow-sorted AMs isolated from KO mice (Figure 3A). Compared to gene expression at 4 weeks, there were 671 genes at 8-week, 938 genes at 12-week, and 1344 genes at 40-week that were significantly up-regulated (Figures 3B–D, FDR ≤ 0.05). The Venn diagram indicated that expression of 153 genes was remarkably altered at all time-points in comparison to that at 4-week (Figure 3E). The ten most significantly up-regulated and down-regulated genes along with FDR values are presented in Supplementary Table 3.

To explore the biomedical significance of KO expressed genes, we analyzed the pathways by using GAGE method and screening through GO and KEGG databases (Figures 3F–I). Interestingly, there was no significant change in GO molecular function pathway among the KO age groups (Supplementary Table 4). On the other hand, 18 and 6 GO biological processes were significantly up-regulated at 8 and 12 weeks of age, respectively, compared to that at 4 weeks. At 40-week, no significant change was, however, found in GO biological process. In the KEGG metabolism pathway analysis, antigen processing and presentation, and cell adhesion molecule pathways were significantly up-regulated at 8-week and 12-week than that at 4-week. In the KEGG disease pathway analysis, several infectious disease-related pathways including that of Influenza A, EBV, viral myocarditis, tuberculosis, and Herpes simplex virus (HSV) were found significantly up-regulated at 8, 12, and 40 weeks of age.

In brief, most of the pathways for immunologic and inflammatory responses were activated by 8 weeks and these pathways were less pronounced than that in the WT mice, suggesting an early onset of inflammation in KO mice.

### Differential Expression of Genes and Gene Sets in KO and WT AMs

**FIGURE 4:**
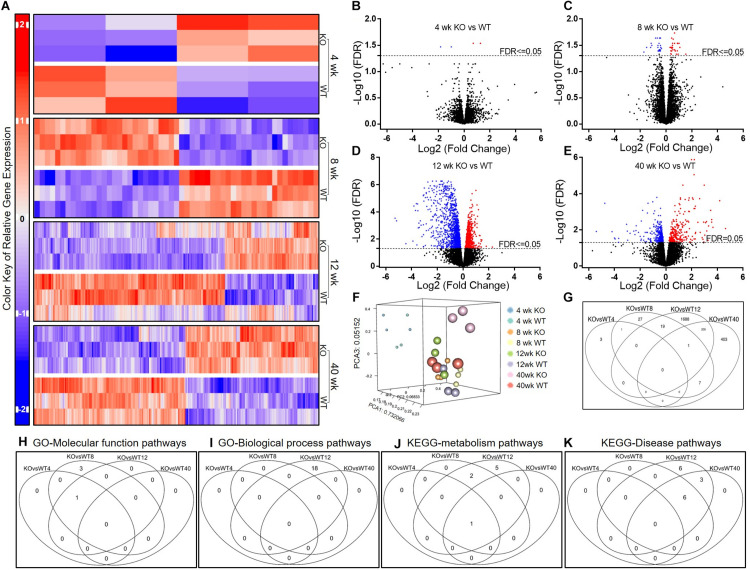
Comparison of the AM gene expression patterns in *Scgb1a1* KO and WT mice. **(A)** Up-regulated and down-regulated genes in AMs at 4, 8, 12, and 40 weeks; **(B–E)**. Volcano plots of significantly up-regulated (red) and down-regulated (blue) genes (FDR < 0.05); **(F)** PCA plot of the variant feature of gene expression; **(G)** Venn diagrams of the significantly changed genes. Venn diagrams of the significantly changed gene sets in AMs from KO vs WT mice at different ages including GO-Molecular function pathways **(H)**, GO-Biological process pathways **(I)**, KEGG-metabolism pathways **(J)**, and KEGG-Disease pathways **(K)**. FDR < 0.05 was considered significant; FC, fold change.

To evaluate the effect of *Scgb1a1* deficiency on AM transcriptome, we compared the gene expression patterns between KO and WT across the age groups (Figure 4A). As shown in Figures 4B–E, 4 genes at 4-week, 55 genes at 8-week, 1913 genes at 12-week, and 616 genes at 40-week were significantly altered in the KO AMs. The PCA plot showed gene expression patterns over time in the KO and WT mice (Figure 4F). The overlap between these genes was summarized in Figure 4G showing that the mice at 12-week had the most significant changes in gene expression. It was further presented in a Venn diagram indicating that 656 genes were remarkably altered at all time-points compared to that at 4-week (Figure 4G). The ten most significantly up-regulated and down-regulated genes are presented in Supplementary Table 5.

To decipher biologic relevance of gene expression patterns, we analyzed the significantly changed gene sets by using GAGE method and screened through GO and KEGG databases (Figures 4H–K). As shown in Supplementary Table 6, Ribosome was the only one GO molecular function pathway that was significantly down-regulated in 4-week old KO mice compared to that in WT. In addition to Ribosome pathway, there were three GO molecular function pathways that were down-regulated in the KO mice at 8 weeks of age. Surprisingly, 17 GO biological processes that are related to immune response or inflammation were significantly down-regulated in the KO at 12-week. From analysis of KEGG metabolism pathways, the antigen processing and presentation, proteasome, TNF signaling, and NOD-like receptor signaling were also significantly down-regulated in KO at 12-week. In KEGG disease pathway analysis, several infectious disease and immune related pathways including that of Influenza A, EBV, HCV, and HSV were significantly down-regulated whereas the non-alcoholic liver disease was significantly up-regulated at 12-week in the KO mice.

In sum, pathways for immunologic and inflammatory responses were less pronounced in KO AMs suggesting that *Scgb1a1* deficiency may undermine the repertoire of immune defenses available to this age group.

### Attenuation of AM-Mediated Inflammation by SCGB1A1

**FIGURE 5:**
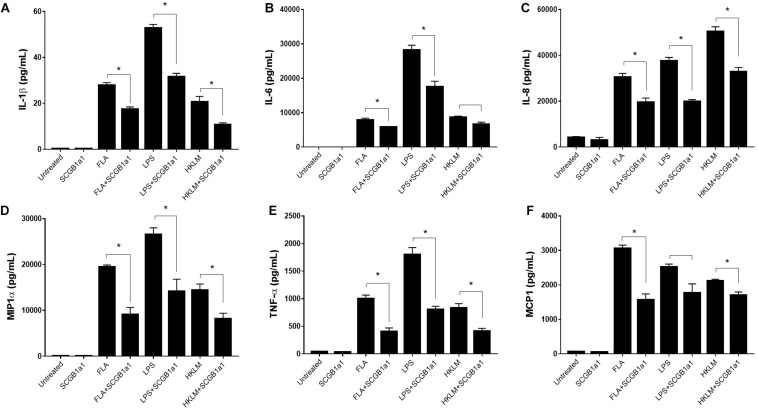
SCGB1A1 attenuates AM-mediated inflammation. Cytokine and chemokine release from purified C57BL/6 AMs in response to toll-like receptor (TLR) agonists were measured: TLR2- heat-killed Listeria monocytogenes (HKLM), TLR4-Lipopolysaccharide from Escherichia coli K12 (LPS), and TLR5- Salmonella typhimurium Flagellin (FLA) in the presence or absence of recombinant SCGB1a1 protein. The panels depict IL-1b **(A)**, IL-6 **(B)**, IL-8 **(C)**, MIP1a **(D)**, TNF-α **(E)**, and MCP1 **(F)** responses to TLR stimuli. Data from three biological replicates are plotted as mean ± SEM, two-tailed unpaired *t*-tests were applied, and *p* < 0.05 was considered significant (*).

We measured the cytokines and chemokines including interleukin (IL)-1β, IL-6, IL-8, MIP-1α, tumor necrosis factor (TNF)-α, and MCP-1 released by the flow-sorted AMs from C57BL/6 mice in response to TLR agonists: TLR2- heat-killed Listeria monocytogenes (HKLM), TLR4-Lipopolysaccharide from *Escherichia coli* K12 (LPS) and TLR5- *Salmonella typhimurium* Flagellin (FLA) in presence or absence of recombinant SCGB1a1 protein. As shown in Figure 5A, there was no significant change in the IL-1β level in the SCGB1a1 treated AMs (0.233 ± 0.033 pg/mL) than that in the untreated AMs (0.167 ± 0.120 pg/mL). However, the IL-1β release by AM was strikingly high when AMs were stimulated by LPS (52.660 ± 1.719 pg/mL, *p* < 0.001), HKLM (20.613 ± 2.426 pg/mL, *p* = 0.001), or FLA (27.707 ± 1.304 pg/mL, *p* < 0.001) than that in the untreated group. Moreover, the TLR stimulated cytokine/chemokine release was significantly reduced by supplementation of SCGB1a1. The IL-1β levels in LPS + SCGB1a1 (31.490 ± 1.588 pg/mL, *p* < 0.001), HKLM + SCGB1a1 (10.673 ± 0.876 pg/mL, *p* = 0.001), or FLA + SCGB1a1 (17.347 ± 1.130 pg/mL, *p* = 0.001) treated AMs were significantly lower than that the LPS, HKLM, or FLA only treated AMs. Furthermore, the administration of SCGB1a1 consistently lowered IL-6, IL-8, MIP-1α, TNF-α, and MCP-1 release from TLR induced AMs (Figures 5B–F). These data indicated that SCGB1a1 protein supplementation reduced the cytokine release induced by various microbial stimuli.

## Discussion

Alveolar macrophages are long-lived lung-resident phagocytes that occur in the alveoli up to 6 × 10^9^ cells per healthy human adult (54) and are known to persist multitudes of infection, inflammation and autoimmune conditions. While contribution of AMs in eliciting pathogen and self-antigen specific immune responses (14–17) have been well recognized, their role in the inflammatory complications has not been fully understood. To investigate potential anomalies in AM development and function in the absence of SCGB1A1 protein, we studied the phenotypes of AMs in age and gender matched *Scgb1a1*^–/–^ and *Scgb1a1*^+/+^ mice. While vast majority (>90%) of AMs were identified as CD11c^hi^ and Siglec-F^hi^ leukocytes in BAL cells, a minor population exhibited a phenotypic variation to be CD11c^hi^ and Siglec-F^*low*^ consisting of 6.85% in the KO compared to 0.96% that in the WT. Developmental origin and pathogenic significance of the CD11c^hi^ and Siglec-F^*low*^ cells in *Scgb1a1*^–/–^ are currently unknown; however, some unrelated studies have suggested inflammatory monocytes as precursor for the Siglec-F^*low*^ pulmonary macrophages (52, 53). This phenotypic difference in AMs as early as 12 weeks of age (early adult) suggests a variation in the myelopoiesis and/or activation status associated with *Scgb1a1* deficiency. Furthermore, purified AMs from WT and *Scgb1a1*^–/–^ KO mice were studied by RNA sequencing to capture their transcriptome profiles in a time dependent manner.

Our study reveals a progressive AM transcriptome from the *Scgb1a1* WT mice at weaning (4 week), puberty (8 week), early adult (12 week), and middle age (40 week) whereas that in *Scgb1a1* deficiency resembles the disorder of SCGB1A1 depletion induced by smoking, COPD and other types acute and chronic lung injuries in the humans. Specifically, in the WT mice, we found that the biological process pathways involving antigen presentation, innate immune and anti-viral defenses were significantly up-regulated at puberty, early adult, and middle age than that at weaning. Strikingly, WT mice at the early adult age had the most up-regulated immunological pathways suggesting that this age group can harness higher anti-viral immunity mediated by AMs. Although an increase in these processes was observed in the KO mice with most activated pathways found at puberty that further declined at early adult age and beyond, this indicated a premature and weakened immune system in the KO mice. These immunological changes between the KO and WT mice were also observed when analyzed by an age-paired comparison where the KO mice, at early adult age, had the most compromised immune system than that in the WT mice. These dynamic changes in anti-viral immunity during AM development may have a clinical relevance on the varied outcomes of COVID-19 among stratified age groups.

Both of the SARS-COV causing SARS (55) and SARS-COV-2 causing COVID-19 (56) are known to utilize angiotensin-converting enzyme 2 (ACE2) as a cell attachment receptor and entry site. Hence, the tissue distribution of ACE2 and its dynamic expression are crucial to understand the pathophysiology of coronavirus infection. It has been shown that ACE2 is expressed in numerous tissues, including epithelial cells of the lung, intestine, kidney and blood vessels (56). Within the lung, the ACE2 is heavily expressed on alveolar type (AT) II cells (57–59) and it is also found on the SCGB1A1-producing club cells (60) that make these cell types vulnerable to the coronavirus infection. This is particularly important since COVID-19 is an airborne disease and SARS-COV-2 can travel through the inhaled air to the permissive sites of infection in respiratory bronchioles and alveoli. It is also concerning that smoking up-regulates ACE2 expression in lungs (61) and a recent study found current smokers to be 120% more likely to die from SARS-COV-2 infection than non-smokers (4). The human AMs are also susceptible to a strain of coronavirus infection (62). Together, these findings highlight the importance of AMs and club cells in the possible entry and transmission of coronaviruses.

Anti-inflammatory effector function of SCGB1A1 has been well studied and it has been shown that viral and bacterial infections of lungs deficient in *Scgb1a1* elicit greater inflammatory responses. The pro-inflammatory cytokine concentration and BAL cell count were significantly higher in S*cgb1a1*-deficient lungs following an acute Adenovirus infection (34). Similarly, infection by Respiratory syncytial virus led to increased T-helper 2 cytokines, neutrophil chemokines and viral replication following *Scgb1a1* deficiency whereas restoration of *Scgb1a1* expression in the airway abrogated the viral persistence and lung inflammation (33). It has been reported that club cells from mouse airways can modulate cytokine production by macrophages in the lung periphery (63). Another *in vitro* study also found that supplementation of exogenous SCGB1a1 can reverse cigarette smoke-induced IL-8 release and attenuate airway inflammation in biopsy specimens from patients with COPD (64). Currently, COVID-19 is treated with supportive care and respiratory failure from acute respiratory distress syndrome is the leading cause of death (65). Accumulating evidence suggests that a subgroup of patients with severe COVID-19 might have cytokine storm syndrome (5) indicating an urgent need for additional intervention and/or prophylaxis beyond supportive care. In the present study, we found exogenous supplementation of SCGB1a1 protein significantly blunted AM release of cytokine storm syndrome mediators including IL-6, IL-1β, TNF-α, IL-8, MIP-1α, and MCP-1. Moreover, AMs are critical in eliciting anti-coronavirus CD4^+^ T cells that remain important in mounting a specific and lasting immune defense (66). Cytokine storm is believed to dampen AM’s antigen presentation ability and cytokine neutralization results in a greater frequency of anti-viral T cells. Therefore, implementation of an early curb on AM inflammation and cytokine surge may produce better outcome measures in managing COVID-19 with lower mortality and higher virus specific immunity.

Although findings from this study are interesting and likely to have a high clinical relevance, there are several limitations in the study. For instance, the study did not assess bi-directional interactions between AMs and club cells, and their influence on lung tissue repair, remodeling, and regeneration. Such crosstalk may provide important insights into pulmonary health and serve as early indicators of AM activation and/or club cell damage under physiologic and pathologic conditions. Additionally, we did not evaluate the association of SCGB1A1 concentration with ACE2 expression in AT II cells. Due to current non-availability of humanized ACE2 transgenic mouse model, we were unable to perform *in vivo* infection studies with SARS-CoV-2 in the setting of *Scgb1a1* deficiency.

**FIGURE 6:**
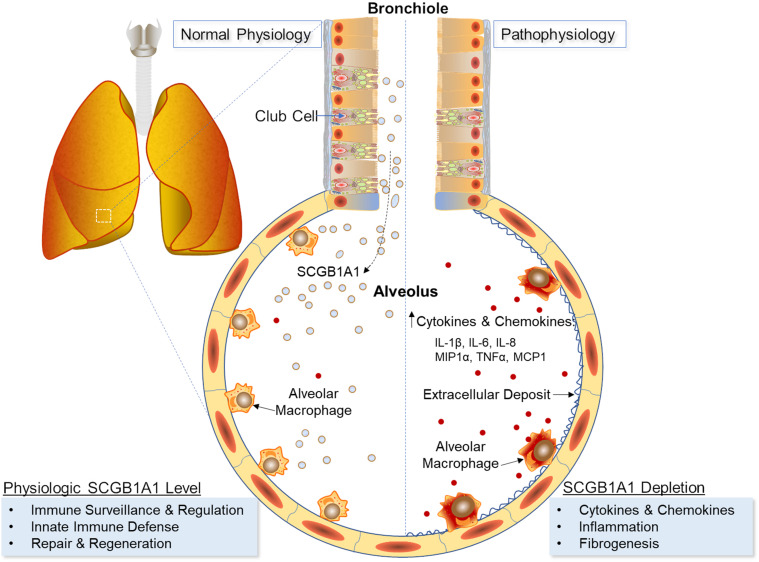
Schematic representation of SCGB1A1 in AM-mediated inflammation and immunity.

In sum, SCGB1A1 influenced AM functionality where AMs developing under Scgb1a1 deficiency showed a diminished ability to stimulate adaptive immune responses. Given the high density of AMs in lung tissue, exogenous supplementation of SCGB1A1 may be helpful to restore AM SteadyState functions and prevent local cytokine surges in infectious and autoimmune diseases (Figure 6).

## Conclusion

Although AMs and lung surfactants have been widely studied, their respective roles have been analyzed more in isolation than in tandem. To our knowledge, this is the first investigation that establishes a functional link between these two entities where Scgb1a1, a constituent of the lung surfactant, regulates AM development, gene transcription, and responsiveness to inflammation. In general, AMs developing under Scgb1a1-deficiency were skewed toward an inflammatory phenotype, whereas exogenous supplementation of recombinant SCGB1A1 protein exhibited anti-inflammatory effects on AM activation. While it is imperative to maintain physiologic levels of SCGB1A1 in the lung milieu for an optimal SteadyState respiration, we speculate that a lung locale overexpression of Scgb1a1 via gene therapy or an airway delivery of recombinant protein might be helpful in curbing lung inflammation and cytokine surge in the management of COVID-19 and other inflammatory lung diseases.

## Data Availability Statement

The datasets presented in this study can be found in the Gene Expression Omnibus (GEO) repository with accession number GSE148647 (https://www.ncbi.nlm.nih.gov/geo/query/acc.cgi?acc=GSE148647).

## Ethics Statement

The animal study was reviewed and approved by Institutional Animal Care and Use Committee, Washington University in St. Louis School of Medicine, St. Louis, MO, United States.

## Author Contributions

MX performed data analysis and manuscript writing. WY performed sequence data analysis and interpretation. XW reviewed the manuscript. DN designed the study, and performed data analysis and manuscript writing and editing. All authors contributed to the article and approved the submitted version.

## Conflict of Interest

The authors declare that the research was conducted in the absence of any commercial or financial relationships that could be construed as a potential conflict of interest.

## References

[1] University JH. *Johns Hopkins Experts in Global Public Health, Infectious Disease, and Emergency Preparedness Have Been at the Forefront of the International Response to COVID-19.* Baltimore, MD: Johns Hopkins (2020).

[2] Dong E Du H Gardner L. An interactive web-based dashboard to track COVID-19 in real time. *Lancet Infect Dis.* (2020) 20:533–4. 10.1016/S1473-3099(20)30120-1 32087114 PMC7159018

[3] Guan WJ Ni ZY Hu Y Liang WH Ou CQ He JX Clinical characteristics of coronavirus disease 2019 in China. *New Engl J Med.* (2020) 382:1708–20. 10.1056/NEJMoa2002032 PMC7092819 32109013

[4] Zhou F Yu T Du R Fan G Liu Y Liu Z Clinical course and risk factors for mortality of adult inpatients with COVID-19 in Wuhan, China: a retrospective cohort study. *Lancet (Lond Engl).* (2020) 395:1054–62. 10.1016/S0140-6736(20)30566-3 PMC7270627 32171076

[5] Mehta P McAuley DF Brown M Sanchez E Tattersall RS Manson JJ. COVID-19: consider cytokine storm syndromes and immunosuppression. *Lancet (Lond Engl).* (2020) 395:1033–4. 10.1016/S0140-6736(20)30628-0 PMC7270045 32192578

[6] Perdiguero GE Klapproth K Schulz C Busch K Azzoni E Crozet L Tissue-resident macrophages originate from yolk-sac-derived erythro-myeloid progenitors. *Nature.* (2015) 518:547–51. 10.1038/nature13989 25470051 PMC5997177

[7] Guilliams M De Kleer I Henri S Post S Vanhoutte L De Prijck S Alveolar macrophages develop from fetal monocytes that differentiate into long-lived cells in the first week of life via GM-CSF. *J Exp Med.* (2013) 210:1977–92. 10.1084/jem.20131199 24043763 PMC3782041

[8] Cardani A Boulton A Kim TS Braciale TJ. Alveolar macrophages prevent lethal influenza pneumonia by inhibiting infection Of Type-1 alveolar epithelial cells. *PLoS Pathog.* (2017) 13:e1006140. 10.1371/journal.ppat.1006140 28085958 PMC5268648

[9] Ghoneim HE Thomas PG McCullers JA. Depletion of alveolar macrophages during influenza infection facilitates bacterial superinfections. *J Immunol.* (2013) 191:1250–9. 10.4049/jimmunol.1300014 23804714 PMC4907362

[10] MacLean JA Xia W Pinto CE Zhao L Liu HW Kradin RL. Sequestration of inhaled particulate antigens by lung phagocytes. A mechanism for the effective inhibition of pulmonary cell-mediated immunity. *Am J Pathol.* (1996) 148:657–66. 8579128 PMC1861667

[11] Nakamura T Abu-Dahab R Menger MD Schafer U Vollmar B Wada H Depletion of alveolar macrophages by clodronate-liposomes aggravates ischemia-reperfusion injury of the lung. *J Heart Lung Transplant.* (2005) 24:38–45. 10.1016/j.healun.2003.10.007 15653377

[12] Schneider C Nobs SP Heer AK Kurrer M Klinke G van Rooijen N Alveolar macrophages are essential for protection from respiratory failure and associated morbidity following influenza virus infection. *PLoS Pathog.* (2014) 10:e1004053. 10.1371/journal.ppat.1004053 24699679 PMC3974877

[13] Allard B Panariti A Martin JG. Alveolar macrophages in the resolution of inflammation, tissue repair, and tolerance to infection. *Front Immunol.* (2018) 9:1777. 10.3389/fimmu.2018.01777 30108592 PMC6079255

[14] Macdonald DC Singh H Whelan MA Escors D Arce F Bottoms SE Harnessing alveolar macrophages for sustained mucosal T-cell recall confers long-term protection to mice against lethal influenza challenge without clinical disease. *Mucosal Immunol.* (2014) 7:89–100. 10.1038/mi.2013.27 23715172

[15] Benoit A Huang Y Proctor J Rowden G Anderson R. Effects of alveolar macrophage depletion on liposomal vaccine protection against respiratory syncytial virus (RSV). *Clin Exp Immunol.* (2006) 145:147–54. 10.1111/j.1365-2249.2006.03114.x 16792685 PMC1941998

[16] Nayak DK Zhou F Xu M Huang J Tsuji M Yu J Zbtb7a induction in alveolar macrophages is implicated in anti-HLA-mediated lung allograft rejection. *Sci Transl Med.* (2017) 10.1126/scitranslmed.aal1243 9:398. 28701473 PMC5846477

[17] Nayak DK Zhou F Xu M Huang J Tsuji M Hachem R Long-term persistence of donor alveolar macrophages in human lung transplant recipients that influences donor specific immune responses. *Am J Transplant.* (2016) 16:2300–11. 10.1111/ajt.13819 27062199 PMC5289407

[18] Losa García JE Rodríguez FM Martín de Cabo MR. García SMJ Losada JP Villarón LG Evaluation of inflammatory cytokine secretion by human alveolar macrophages. *Mediators Inflamm.* (1999) 8:43–51. 10.1080/09629359990711 10704089 PMC1781780

[19] Reynolds SD Malkinson AM. Clara cell: progenitor for the bronchiolar epithelium. *Int J Biochem Cell Biol.* (2010) 42:1–4. 10.1016/j.biocel.2009.09.002 19747565 PMC2787899

[20] Zheng D Limmon GV Yin L Leung NH Yu H Chow VT A cellular pathway involved in Clara cell to alveolar type II cell differentiation after severe lung injury. *PLoS One.* (2013) 8:e71028. 10.1371/journal.pone.0071028 23940685 PMC3734298

[21] Zheng D Yin L Chen J. Evidence for Scgb1a1(+) cells in the generation of p63(+) cells in the damaged lung parenchyma. *Am J Respir Cell Mol Biol.* (2014) 50:595–604. 10.1165/rcmb.2013-0327OC 24134540

[22] Zheng D Soh BS Yin L Hu G Chen Q Choi H Differentiation of club cells to alveolar epithelial cells in vitro. *Sci Rep.* (2017) 7:41661. 10.1038/srep41661 28128362 PMC5269679

[23] Reynolds SD Hong KU Giangreco A Mango GW Guron C Morimoto Y Conditional clara cell ablation reveals a self-renewing progenitor function of pulmonary neuroendocrine cells. *Am J Physiol Lung Cell Mol Physiol.* (2000) 278:L1256–63. 10.1152/ajplung.2000.278.6.L1256 10835332

[24] Mukherjee AB Zhang Z Chilton BS. Uteroglobin: a steroid-inducible immunomodulatory protein that founded the Secretoglobin superfamily. *Endocrine Rev.* (2007) 28:707–25. 10.1210/er.2007-0018 17916741

[25] Mandal AK Ray R Zhang Z Chowdhury B Pattabiraman N Mukherjee AB. Uteroglobin inhibits prostaglandin F2alpha receptor-mediated expression of genes critical for the production of pro-inflammatory lipid mediators. *J Biol Chem.* (2005) 280:32897–904. 10.1074/jbc.M502375200 16061484

[26] Mandal AK Zhang Z Ray R Choi MS Chowdhury B Pattabiraman N Uteroglobin represses allergen-induced inflammatory response by blocking PGD2 receptor-mediated functions. *J Exp Med.* (2004) 199:1317–30. 10.1084/jem.20031666 15148333 PMC2211805

[27] Chowdhury B Mantile-Selvaggi G Miele L Cordella-Miele E Zhang Z Mukherjee AB. Lys 43 and Asp 46 in alpha-helix 3 of uteroglobin are essential for its phospholipase A2 inhibitory activity. *Biochem Biophys Res Commun.* (2002) 295:877–83. 10.1016/S0006-291X(02)00767-2 12127976

[28] Okutani R Itoh Y Yamada T Yamaguchi T Singh G Yagisawa H Preparation and characterization of human recombinant protein 1/Clara cell M(r) 10,000 protein. *Eur J Clin Chem Clin Biochem.* (1996) 34:691–6. 10.1515/cclm.1996.34.9.691 8891520

[29] Lesur O Bernard A Arsalane K Lauwerys R Bégin R Cantin A Clara cell protein (CC-16) induces a phospholipase A2-mediated inhibition of fibroblast migration in vitro. *Am J Respir Crit Care Med.* (1995) 152:290–7. 10.1164/ajrccm.152.1.7541278 7541278

[30] Long XB Hu S Wang N Zhen HT Cui YH Liu Z. Clara cell 10-kDa protein gene transfection inhibits NF-κB activity in airway epithelial cells. *PLoS One.* (2012) 7:e35960. 10.1371/journal.pone.0035960 22558282 PMC3338482

[31] Pang M Wang H Bai JZ Cao D Jiang Y Zhang C Recombinant rat CC16 protein inhibits LPS-induced MMP-9 expression via NF-κB pathway in rat tracheal epithelial cells. *Exp Biol Med (Maywood).* (2015) 240:1266–78. 10.1177/1535370215570202 25716019 PMC4935251

[32] Matsumoto T Fujita M Hirano R Uchino J Tajiri Y Fukuyama S Chronic. *Int J Chron Obstruct Pulmon Dis.* (2016) 11:2321–7. 27703342 10.2147/COPD.S113707 PMC5036550

[33] Wang SZ Rosenberger CL Bao YX Stark JM Harrod KS. Clara cell secretory protein modulates lung inflammatory and immune responses to respiratory syncytial virus infection. *J Immunol.* (2003) 171:1051–60. 10.4049/jimmunol.171.2.1051 12847279

[34] Harrod KS Mounday AD Stripp BR Whitsett JA. Clara cell secretory protein decreases lung inflammation after acute virus infection. *Am J Physiol.* (1998) 275(5 Pt 1):L924–30. 10.1152/ajplung.1998.275.5.L924 9815110

[35] Kropski JA Fremont RD Calfee CS Ware LB. Clara cell protein (CC16), a marker of lung epithelial injury, is decreased in plasma and pulmonary edema fluid from patients with acute lung injury. *Chest.* (2009) 135:1440–7. 10.1378/chest.08-2465 19188556 PMC2716712

[36] Bernard AM Gonzalez-Lorenzo JM Siles E Trujillano G Lauwerys R. Early decrease of serum Clara cell protein in silica-exposed workers. *Eur Respir J.* (1994) 7:1932–7. 7875262

[37] Zhu L Di PY Wu R Pinkerton KE Chen Y. Repression of CC16 by cigarette smoke (CS) exposure. *PLoS One.* (2015) 10:e0116159. 10.1371/journal.pone.0116159 25635997 PMC4312097

[38] Blomberg A Mudway I Svensson M Hagenbjork-Gustafsson A Thomasson L Helleday R Clara cell protein as a biomarker for ozone-induced lung injury in humans. *Eur Respir J.* (2003) 22:883–8. 10.1183/09031936.03.00048203 14680073

[39] Bourdin A Mifsud NA Chanez B McLean C Chanez P Snell G Donor clara cell secretory protein polymorphism is a risk factor for bronchiolitis obliterans syndrome after lung transplantation. *Transplantation.* (2012) 94:652–8. 10.1097/TP.0b013e31825ffca6 22902791

[40] Kelly FL Kennedy VE Jain R Sindhwani NS Finlen Copeland CA Snyder LD Epithelial clara cell injury occurs in bronchiolitis obliterans syndrome after human lung transplantation. *Am J Transplant.* (2012) 12:3076–84. 10.1111/j.1600-6143.2012.04201.x 22883104 PMC3484196

[41] Shah RJ Wickersham N Lederer DJ Palmer SM Cantu E Diamond JM Preoperative plasma club (clara) cell secretory protein levels are associated with primary graft dysfunction after lung transplantation. *Am J Transplant.* (2014) 14:446–52. 10.1111/ajt.12541 24400993 PMC3946770

[42] Nomori H Horio H Fuyuno G Kobayashi R Morinaga S Hirabayashi Y. Protein 1 (Clara cell protein) serum levels in healthy subjects and patients with bacterial pneumonia. *Am J Respir Crit Care Med.* (1995) 152:746–50. 10.1164/ajrccm.152.2.7633737 7633737

[43] Kurowski M Jurczyk J Jarzebska M Moskwa S Makowska JS Krysztofiak H Association of serum Clara cell protein CC16 with respiratory infections and immune response to respiratory pathogens in elite athletes. *Respirat Res.* (2014) 15:45. 10.1186/1465-9921-15-45 24735334 PMC3997232

[44] Shijubo N Itoh Y Yamaguchi T Sugaya F Hirasawa M Yamada T Serum levels of Clara cell 10-kDa protein are decreased in patients with asthma. *Lung.* (1999) 177:45–52. 10.1007/PL00007626 9835633

[45] Lensmar C Nord M Gudmundsson GH Roquet A Andersson O Jörnvall H Decreased pulmonary levels of the anti-inflammatory Clara cell 16 kDa protein after induction of airway inflammation in asthmatics. *Cell Mol Life Sci.* (2000) 57:976–81. 10.1007/PL00000738 10950311 PMC11147083

[46] Yoshikawa S Miyahara T Reynolds SD Stripp BR Anghelescu M Eyal FG Clara cell secretory protein and phospholipase A2 activity modulate acute ventilator-induced lung injury in mice. *J Appl Physiol.* (2005) 98:1264–71. 10.1152/japplphysiol.01150.2004 15608088

[47] Westphalen K Gusarova GA Islam MN Subramanian M Cohen TS Prince AS Sessile alveolar macrophages communicate with alveolar epithelium to modulate immunity. *Nature.* (2014) 506:503–6. 10.1038/nature12902 24463523 PMC4117212

[48] Zhang Z Kundu GC Yuan CJ Ward JM Lee EJ DeMayo F Severe fibronectin-deposit renal glomerular disease in mice lacking uteroglobin. *Science (New York N Y).* (1997) 276:1408–12. 10.1126/science.276.5317.1408 9162006

[49] Dutta S Sengupta P. Men and mice: relating their ages. *Life Sci.* (2016) 152:244–8. 10.1016/j.lfs.2015.10.025 26596563

[50] Nayak DK Mendez O Bowen S Mohanakumar T. Isolation and in vitro culture of murine and human alveolar macrophages. *J Vis Exp.* (2018) 134:57287. 10.3791/57287 29733312 PMC6100701

[51] Luo W Friedman MS Shedden K Hankenson KD Woolf PJ. GAGE: generally applicable gene set enrichment for pathway analysis. *BMC Bioinformat.* (2009) 10:161. 10.1186/1471-2105-10-161 19473525 PMC2696452

[52] Misharin AV Morales-Nebreda L Reyfman PA Cuda CM Walter JM McQuattie-Pimentel AC Monocyte-derived alveolar macrophages drive lung fibrosis and persist in the lung over the life span. *J Exp Med.* (2017) 4:2387–404. 10.1084/jem.20162152 PMC5551573 28694385

[53] Zheng Z Chiu S Akbarpour M Sun H Reyfman PA Anekalla KR Donor pulmonary intravascular nonclassical monocytes recruit recipient neutrophils and mediate primary lung allograft dysfunction. *Sci Transl Med.* (2017) 9:394. 10.1126/scitranslmed.aal4508 28615357 PMC5568853

[54] Stone KC Mercer RR Gehr P Stockstill B Crapo JD. Allometric relationships of cell numbers and size in the mammalian lung. *Am J Respir Cell Mol Biol.* (1992) 6:235–43. 10.1165/ajrcmb/6.2.235 1540387

[55] Li W Moore MJ Vasilieva N Sui J Wong SK Berne MA Angiotensin-converting enzyme 2 is a functional receptor for the SARS coronavirus. *Nature.* (2003) 426:450–4. 10.1038/nature02145 14647384 PMC7095016

[56] Hoffmann M Kleine-Weber H Schroeder S Krüger N Herrler T Erichsen S SARS-CoV-2 cell entry depends on ACE2 and TMPRSS2 and is blocked by a clinically proven protease inhibitor. *Cell.* (2020) 181:271–80.e8. 10.1016/j.cell.2020.02.052 32142651 PMC7102627

[57] Ding Y He L Zhang Q Huang Z Che X Hou J Organ distribution of severe acute respiratory syndrome (SARS) associated coronavirus (SARS-CoV) in SARS patients: implications for pathogenesis and virus transmission pathways. *J Pathol.* (2004) 203:622–30. 10.1002/path.1560 15141376 PMC7167761

[58] Hamming I Timens W Bulthuis ML Lely AT Navis G van Goor H. Tissue distribution of ACE2 protein, the functional receptor for SARS coronavirus. A first step in understanding SARS pathogenesis. *J Pathol.* (2004) 203:631–7. 10.1002/path.1570 15141377 PMC7167720

[59] He L Ding Y Zhang Q Che X He Y Shen H Expression of elevated levels of pro-inflammatory cytokines in SARS-CoV-infected ACE2+ cells in SARS patients: relation to the acute lung injury and pathogenesis of SARS. *J Pathol.* (2006) 210:288–97. 10.1002/path.2067 17031779 PMC7167655

[60] Wiener RS Cao YX Hinds A Ramirez MI Williams MC. Angiotensin converting enzyme 2 is primarily epithelial and is developmentally regulated in the mouse lung. *J Cell Biochem.* (2007) 101:1278–91. 10.1002/jcb.21248 17340620 PMC7166549

[61] Brake S Barnsley K Lu W McAlinden K Eapen M Sohal S. Smoking upregulates angiotensin-converting enzyme-2 receptor: a potential adhesion site for novel coronavirus SARS-CoV-2 (Covid-19). *J Clin Med.* (2020) 9:841. 10.3390/jcm9030841 32244852 PMC7141517

[62] Funk CJ Wang J Ito Y Travanty EA Voelker DR Holmes KV Infection of human alveolar macrophages by human coronavirus strain 229E. *J Gen Virol.* (2012) 93(Pt 3):494–503. 10.1099/vir.0.038414-0 22090214 PMC3352353

[63] Elizur A Adair-Kirk TL Kelley DG Griffin GL deMello DE Senior RM. Clara cells impact the pulmonary innate immune response to LPS. *Am J Physiol Lung Cell Mol Physiol.* (2007) 293:L383–92. 10.1152/ajplung.00024.2007 17526599

[64] Gamez AS Gras D Petit A Knabe L Molinari N Vachier I Supplementing defect in club cell secretory protein attenuates airway inflammation in COPD. *Chest.* (2015) 147:1467–76. 10.1378/chest.14-1174 25474370

[65] Ruan Q Yang K Wang W Jiang L Song J. Clinical predictors of mortality due to COVID-19 based on an analysis of data of 150 patients from Wuhan, China. *Intens Care Med.* (2020) 46:846–8. 10.1007/s00134-020-05991-x 32125452 PMC7080116

[66] Hartwig SM Holman KM Varga SM. Depletion of alveolar macrophages ameliorates virus-induced disease following a pulmonary coronavirus infection. *PLoS One.* (2014) 9:e90720. 10.1371/journal.pone.0090720 24608125 PMC3946553

