# Supplementary material for: Lung Secretoglobin Scgb1a1 Influences Alveolar Macrophage-Mediated Inflammation and Immunity
Source: Front Immunol. 2020 Oct 1;11:584310. doi: 10.3389/fimmu.2020.584310 (PMC7558713; doi:10.3389/fimmu.2020.584310)
Supplement: Supplementary file 1 [file Data_Sheet_1.pdf]

Supplementary Material

Supplementary Tables

Table S1: Ten most significantly upregulated and downregulated genes in AMs from wild type (WT) mice.

Comparison (WT time course)	Gene name	Gene expression	FDR	Gene description
8wks vs 4wks	*Hmcn1*	Upregulation	1.56E-05	hemicentin 1 [Source:MGI Symbol;Acc:MGI:2685047]
	Ggt1		1.79E-04	gamma-glutamyltransferase 1 [Source:MGI Symbol;Acc:MGI:95706]
	C1qc		3.82E-03	complement component 1, q subcomponent, C chain [Source:MGI Symbol;Acc:MGI:88225]
	Cd209a		1.79E-04	CD209a antigen [Source:MGI Symbol;Acc:MGI:2157942]
	Heph		1.14E-03	hephaestin [Source:MGI Symbol;Acc:MGI:1332240]
	Lipn		1.32E-03	lipase, family member N [Source:MGI Symbol;Acc:MGI:1917416]
	Ighm		2.33E-02	immunoglobulin heavy constant mu [Source:MGI Symbol;Acc:MGI:96448]
	AI427809		1.12E-03	expressed sequence AI427809 [Source:MGI Symbol;Acc:MGI:2140270]
	Fam19a5		1.78E-03	family with sequence similarity 19, member A5 [Source:MGI Symbol;Acc:MGI:2146182]
	Osbpl5		3.99E-04	oxysterol binding protein-like 5 [Source:MGI Symbol;Acc:MGI:1930265]
	Gm26742	Downregulation	2.29E-05	predicted gene, 26742 [Source:MGI Symbol;Acc:MGI:5477236]
	Gm3235		7.11E-03	predicted gene 3235 [Source:MGI Symbol;Acc:MGI:3781413]
	Arhgap29		2.07E-03	Rho GTPase activating protein 29 [Source:MGI Symbol;Acc:MGI:2443818]
	Spink2		2.01E-03	serine peptidase inhibitor, Kazal type 2 [Source:MGI Symbol;Acc:MGI:1917232]
	Sash1		1.57E-02	SAM and SH3 domain containing 1 [Source:MGI Symbol;Acc:MGI:1917347]
	Plxnb1		9.20E-03	plexin B1 [Source:MGI Symbol;Acc:MGI:2154238]
	Smad6		1.26E-03	SMAD family member 6 [Source:MGI Symbol;Acc:MGI:1336883]
	Lrrtm2		3.15E-02	leucine rich repeat transmembrane neuronal 2 [Source:MGI Symbol;Acc:MGI:2389174]
	Fmr1nb		4.22E-02	Fmr1 neighbor [Source:MGI Symbol;Acc:MGI:2672032]
	Igf2bp3		1.86E-05	insulin-like growth factor 2 mRNA binding protein 3 [Source:MGI Symbol;Acc:MGI:1890359]
12wks vs 4 wks	*Ggt1*	Upregulation	6.72E-05	gamma-glutamyltransferase 1 [Source:MGI Symbol;Acc:MGI:95706]
	Heph		2.25E-04	hephaestin [Source:MGI Symbol;Acc:MGI:1332240]
	Cd209a		5.83E-05	CD209a antigen [Source:MGI Symbol;Acc:MGI:2157942]
	Pla2g2d		4.48E-02	phospholipase A2, group IID [Source:MGI Symbol;Acc:MGI:1341796]
	Slamf8		1.18E-03	SLAM family member 8 [Source:MGI Symbol;Acc:MGI:1921998]
	Fam19a5		4.67E-04	family with sequence similarity 19, member A5 [Source:MGI Symbol;Acc:MGI:2146182]
	Hmcn1		3.85E-04	hemicentin 1 [Source:MGI Symbol;Acc:MGI:2685047]
	Sstr5		1.06E-03	somatostatin receptor 5 [Source:MGI Symbol;Acc:MGI:894282]
	Tlr1		9.17E-04	toll-like receptor 1 [Source:MGI Symbol;Acc:MGI:1341295]
	Fam20c		3.12E-04	family with sequence similarity 20, member C [Source:MGI Symbol;Acc:MGI:2136853]
	Tln2	Downregulation	2.26E-02	talin 2 [Source:MGI Symbol;Acc:MGI:1917799]
	Gm26584		2.08E-02	predicted gene, 26584 [Source:MGI Symbol;Acc:MGI:5477078]
	Gm15530		3.24E-03	predicted gene 15530 [Source:MGI Symbol;Acc:MGI:3782978]
	Cspg4		6.34E-03	chondroitin sulfate proteoglycan 4 [Source:MGI Symbol;Acc:MGI:2153093]
	Spink2		4.52E-03	serine peptidase inhibitor, Kazal type 2 [Source:MGI Symbol;Acc:MGI:1917232]
	4933400F21Rik		2.04E-02	RIKEN cDNA 4933400F21 gene [Source:MGI Symbol;Acc:MGI:1921653]
	Rab36		1.10E-02	RAB36, member RAS oncogene family [Source:MGI Symbol;Acc:MGI:1924127]
	Sash1		1.57E-02	SAM and SH3 domain containing 1 [Source:MGI Symbol;Acc:MGI:1917347]
	Igf2bp3		4.14E-05	insulin-like growth factor 2 mRNA binding protein 3 [Source:MGI Symbol;Acc:MGI:1890359]
	Nov		2.70E-03	nephroblastoma overexpressed gene [Source:MGI Symbol;Acc:MGI:109185]
40wks vs 4wks	*Hmcn1*	Upregulation	2.41E-05	hemicentin 1 [Source:MGI Symbol;Acc:MGI:2685047]
	Lipn		2.63E-03	lipase, family member N [Source:MGI Symbol;Acc:MGI:1917416]
	Ggt1		4.56E-03	gamma-glutamyltransferase 1 [Source:MGI Symbol;Acc:MGI:95706]
	Heph		1.85E-02	hephaestin [Source:MGI Symbol;Acc:MGI:1332240]
	AI427809		6.07E-03	expressed sequence AI427809 [Source:MGI Symbol;Acc:MGI:2140270]
	Cd209a		9.40E-03	CD209a antigen [Source:MGI Symbol;Acc:MGI:2157942]
	Osbpl5		1.12E-03	oxysterol binding protein-like 5 [Source:MGI Symbol;Acc:MGI:1930265]
	Scd1		4.87E-05	stearoyl-Coenzyme A desaturase 1 [Source:MGI Symbol;Acc:MGI:98239]
	Emp2		1.46E-02	epithelial membrane protein 2 [Source:MGI Symbol;Acc:MGI:1098726]
	Mfsd6		2.08E-05	major facilitator superfamily domain containing 6 [Source:MGI Symbol;Acc:MGI:1922925]
	Pkdcc	Downregulation	1.06E-02	protein kinase domain containing, cytoplasmic [Source:MGI Symbol;Acc:MGI:2147077]
	Cspg4		7.73E-03	chondroitin sulfate proteoglycan 4 [Source:MGI Symbol;Acc:MGI:2153093]
	Gm9725		1.82E-02	predicted gene 9725 [Source:MGI Symbol;Acc:MGI:3646552]
	Spink2		5.83E-03	serine peptidase inhibitor, Kazal type 2 [Source:MGI Symbol;Acc:MGI:1917232]
	Nfatc2		3.66E-02	nuclear factor of activated T cells, cytoplasmic, calcineurin dependent 2 [Source:MGI Symbol;Acc:MGI:102463]
	Smad6		3.13E-03	SMAD family member 6 [Source:MGI Symbol;Acc:MGI:1336883]
	Micall2		2.56E-02	MICAL-like 2 [Source:MGI Symbol;Acc:MGI:2444818]
	Sash1		9.79E-03	SAM and SH3 domain containing 1 [Source:MGI Symbol;Acc:MGI:1917347]
	Nov		6.86E-03	nephroblastoma overexpressed gene [Source:MGI Symbol;Acc:MGI:109185]
	Igf2bp3		3.30E-05	insulin-like growth factor 2 mRNA binding protein 3 [Source:MGI Symbol;Acc:MGI:1890359]

Table S2: Upregulated and downregulated gene sets in WT AMs.

GO: Molecular Function

Comparison (WT)	Pathway_ID	Set Size	Mean Log FC	FDR
8wks vs 4wks	GO:0042277 peptide binding	153	4.129	8.39E-03
	GO:0033218 amide binding	179	3.956	1.27E-02
	GO:0004872 receptor activity	371	3.849	1.54E-02
	GO:0005198 structural molecule activity	309	3.520	3.89E-02
	GO:0008009 chemokine activity	19	3.515	3.89E-02
	GO:0016491 oxidoreductase activity	478	3.389	4.68E-02
	GO:0022891 substrate-specific transmembrane transp	384	3.377	4.68E-02
	GO:0003735 structural constituent of ribosome	143	-4.419	6.90E-03
12wks vs 4wks	GO:0042277 peptide binding	153	3.612	4.84E-02
	GO:0033218 amide binding	179	3.556	4.84E-02
	GO:0004872 receptor activity	371	3.522	4.84E-02
	GO:0003735 structural constituent of ribosome	143	-5.506	4.86E-05
40wks vs 4wks	GO:0003735 structural constituent of ribosome	143	-5.506	1.04E-03

KEGG disease

Comparison (WT)	Pathway_ID	Set Size	Mean Log FC	FDR
8wks vs 4wks	mmu05169 Epstein-Barr virus infection	167	3.865	1.19E-02
	mmu05164 Influenza A	125	3.543	1.19E-02
	mmu05150 Staphylococcus aureus infection	31	2.617	3.54E-02
	mmu05162 Measles	93	2.442	4.69E-02
	mmu05016 Huntington's disease	152	3.381	1.19E-02
	mmu05320 Autoimmune thyroid disease	26	3.330	1.19E-02
	mmu04932 Non-alcoholic fatty liver disease (NAFLD)	127	3.287	1.19E-02
	mmu05330 Allograft rejection	27	3.188	1.19E-02
	mmu04940 Type I diabetes mellitus	30	3.165	1.50E-02
	mmu05332 Graft-versus-host disease	27	2.488	4.69E-02
	mmu05416 Viral myocarditis	48	-2.654	3.51E-02
	mmu05168 Herpes simplex infection	158	-3.117	1.19E-02
12wks vs 4wks	mmu05169 Epstein-Barr virus infection	167	4.202	1.29E-03
	mmu05164 Influenza A	125	3.472	6.41E-03
	mmu05168 Herpes simplex infection	158	3.228	7.88E-03
	mmu05416 Viral myocarditis	48	3.220	7.88E-03
	mmu05150 Staphylococcus aureus infection	31	3.215	9.13E-03
	mmu05162 Measles	93	2.668	2.61E-02
	mmu05167 Kaposi's sarcoma-associated herpesvirus i	149	2.526	3.31E-02
	mmu05203 Viral carcinogenesis	167	2.486	3.31E-02
	mmu05152 Tuberculosis	128	2.426	3.51E-02
	mmu05134 Legionellosis	51	2.320	4.25E-02
	mmu05160 Hepatitis C	116	2.290	4.25E-02
	mmu05145 Toxoplasmosis	79	2.220	4.96E-02
	mmu05330 Allograft rejection	27	3.711	6.41E-03
	mmu05320 Autoimmune thyroid disease	26	3.523	7.88E-03
	mmu04940 Type I diabetes mellitus	30	3.309	7.88E-03
	mmu05332 Graft-versus-host disease	27	3.203	9.13E-03
	mmu05322 Systemic lupus erythematosus	52	2.556	3.31E-02
	mmu05020 Prion diseases	24	2.545	3.51E-02
	mmu05418 Fluid shear stress and atherosclerosis	94	2.363	3.98E-02
	mmu04932 Non-alcoholic fatty liver disease (NAFLD)	127	-3.205	7.88E-03
	mmu05016 Huntington's disease	152	-3.594	6.41E-03
40wks vs 4wks	mmu05169 Epstein-Barr virus infection	167	3.405	2.60E-02
	mmu05164 Influenza A	125	3.253	2.60E-02
	mmu05168 Herpes simplex infection	158	3.185	2.60E-02
	mmu05150 Staphylococcus aureus infection	31	2.726	3.57E-02
	mmu05416 Viral myocarditis	48	2.636	3.57E-02
	mmu05167 Kaposi's sarcoma-associated herpesvirus i	149	2.562	3.60E-02
	mmu05162 Measles	93	2.546	3.57E-02
	mmu05016 Huntington's disease	152	3.294	2.60E-02
	mmu05320 Autoimmune thyroid disease	26	2.971	2.60E-02
	mmu04932 Non-alcoholic fatty liver disease (NAFLD)	127	2.806	3.57E-02
	mmu04940 Type I diabetes mellitus	30	2.796	3.57E-02
	mmu05322 Systemic lupus erythematosus	52	2.469	3.72E-02
	mmu05332 Graft-versus-host disease	27	-2.573	3.57E-02
	mmu05330 Allograft rejection	27	-2.949	2.60E-02

GO: Biological process

Comparison (WT)	Pathway_ID	Set Size	lean Log F	FDR
8wks vs 4wks	GO:0045087 innate immune response	422	4.921	2.51E-03
12wks vs 4wks	GO:0045087 innate immune response	422	6.790	5.51E-08
	GO:0043207 response to external biotic stimulus	469	5.490	3.63E-05
	GO:0051707 response to other organism	469	5.490	3.63E-05
	GO:0098542 defense response to other organism	279	5.466	3.63E-05
	GO:0009607 response to biotic stimulus	495	5.421	3.63E-05
	GO:0002252 immune effector process	443	5.136	1.38E-04
	GO:0034341 response to interferon-gamma	63	4.723	1.44E-03
	GO:0050776 regulation of immune response	426	4.630	2.15E-03
	GO:0001817 regulation of cytokine production	389	4.513	2.15E-03
	GO:0002250 adaptive immune response	227	4.392	2.83E-03
	GO:0009617 response to bacterium	290	4.392	2.83E-03
	GO:0034097 response to cytokine	465	4.366	2.83E-03
	GO:0001816 cytokine production	430	4.253	4.27E-03
	GO:0002443 leukocyte mediated immunity	208	4.202	5.57E-03
	GO:0009615 response to virus	187	4.151	6.57E-03
	GO:0002460 adaptive immune response based on som	169	4.077	6.57E-03
	GO:0051607 defense response to virus	154	4.075	7.99E-03
	GO:0019882 antigen processing and presentation	71	4.066	7.99E-03
	GO:0042742 defense response to bacterium	111	4.035	7.99E-03
	GO:0050778 positive regulation of immune response	337	4.023	9.64E-03
	GO:0035456 response to interferon-beta	36	3.830	9.86E-03
	GO:0001819 positive regulation of cytokine production	262	3.823	1.60E-02
	GO:0002449 lymphocyte mediated immunity	157	3.753	2.19E-02
	GO:0071345 cellular response to cytokine stimulus	393	3.693	2.33E-02
	GO:0050865 regulation of cell activation	331	3.658	2.61E-02
	GO:0048002 antigen processing and presentation of pe	43	3.602	3.02E-02
	GO:0002694 regulation of leukocyte activation	310	3.572	3.25E-02
	GO:0071346 cellular response to interferon-gamma	50	3.571	3.66E-02
	GO:0006954 inflammatory response	348	3.529	4.01E-02
	GO:0045088 regulation of innate immune response	188	3.509	4.11E-02
	GO:0050801 ion homeostasis	376	3.572	3.25E-02
	GO:0019725 cellular homeostasis	466	3.472	4.24E-02
	GO:0098771 inorganic ion homeostasis	346	3.460	4.24E-02
	GO:0006820 anion transport	241	3.421	4.24E-02
	GO:0050867 positive regulation of cell activation	197	3.396	4.59E-02
	GO:0034220 ion transmembrane transport	346	3.379	4.85E-02
	GO:0055080 cation homeostasis	337	3.376	4.85E-02
	GO:0006412 translation	493	-4.102	4.85E-02

KEGG metabolism

Comparison (WT)	Pathway_ID	Set Size	lean Log F	FDR
8wks vs 4wks	mmu04612 Antigen processing and presentation	55	4.090	3.55E-03
	mmu04145 Phagosome	120	4.095	3.55E-03
	mmu04514 Cell adhesion molecules (CAMs)	60	3.690	7.70E-03
	mmu03010 Ribosome	133	-5.213	5.37E-05
12wks vs 4wks	mmu04612 Antigen processing and presentation	55	4.176	3.75E-03
	mmu00100 Steroid biosynthesis	15	3.831	1.42E-02
	mmu04145 Phagosome	120	3.640	1.21E-02
	mmu04514 Cell adhesion molecules (CAMs)	60	3.556	1.42E-02
	mmu03050 Proteasome	44	3.058	4.87E-02
	mmu00190 Oxidative phosphorylation	112	-3.079	4.24E-02
	mmu03010 Ribosome	133	-6.223	3.65E-07
40wks vs 4wks	mmu04612 Antigen processing and presentation	55	3.784	1.51E-02
	mmu04145 Phagosome	120	3.383	3.22E-02
	mmu04514 Cell adhesion molecules (CAMs)	60	3.280	3.22E-02
	mmu00100 Steroid biosynthesis	15	-3.127	4.37E-02
	mmu03010 Ribosome	133	-5.590	9.55E-06

Table S3: Ten most significantly upregulated and downregulated genes in AMs from *Scgb1a1*-knockout (KO) mice.

Comparison (KO time course)	Gene name	Gene expression	FDR	Gene description
8wks vs 4wks	*Uty*	Upregulation	4.38E-03	ubiquitously transcribed tetratricopeptide repeat gene, Y chromosome [Source:MGI Symbol;Acc:MGI:894810]
	Eif2s3y		1.02E-02	eukaryotic translation initiation factor 2, subunit 3, structural gene Y-linked [Source:MGI Symbol;Acc:MGI:1349430]
	Ddx3y		4.15E-03	DEAD (Asp-Glu-Ala-Asp) box polypeptide 3, Y-linked [Source:MGI Symbol;Acc:MGI:1349406]
	Slamf8		2.74E-03	SLAM family member 8 [Source:MGI Symbol;Acc:MGI:1921998]
	Sstr5		2.06E-02	somatostatin receptor 5 [Source:MGI Symbol;Acc:MGI:894282]
	Acod1		1.24E-03	aconitate decarboxylase 1 [Source:MGI Symbol;Acc:MGI:103206]
	Ggt1		1.91E-03	gamma-glutamyltransferase 1 [Source:MGI Symbol;Acc:MGI:95706]
	Cd209a		1.72E-03	CD209a antigen [Source:MGI Symbol;Acc:MGI:2157942]
	Kdm5d		3.98E-03	lysine (K)-specific demethylase 5D [Source:MGI Symbol;Acc:MGI:99780]
	Spic		2.49E-05	Spi-C transcription factor (Spi-1/PU.1 related) [Source:MGI Symbol;Acc:MGI:1341168]
	Zfp979	Downregulation	3.42E-02	zinc finger protein 979 [Source:MGI Symbol;Acc:MGI:2148252]
	Zfp119b		2.36E-02	zinc finger protein 119b [Source:MGI Symbol;Acc:MGI:2385323]
	Gm13369		5.64E-03	predicted gene 13369 [Source:MGI Symbol;Acc:MGI:3651010]
	Tln2		1.78E-02	talin 2 [Source:MGI Symbol;Acc:MGI:1917799]
	Gm28286		2.03E-02	predicted gene 28286 [Source:MGI Symbol;Acc:MGI:5578992]
	Cspg4		3.21E-03	chondroitin sulfate proteoglycan 4 [Source:MGI Symbol;Acc:MGI:2153093]
	Adcy4		4.55E-02	adenylate cyclase 4 [Source:MGI Symbol;Acc:MGI:99674]
	Smad6		3.38E-03	SMAD family member 6 [Source:MGI Symbol;Acc:MGI:1336883]
	Igf2bp3		3.62E-04	insulin-like growth factor 2 mRNA binding protein 3 [Source:MGI Symbol;Acc:MGI:1890359]
	Fhdc1		4.14E-02	FH2 domain containing 1 [Source:MGI Symbol;Acc:MGI:2684972]
12wks vs 4 wks	*Uty*	Upregulation	3.29E-03	ubiquitously transcribed tetratricopeptide repeat gene, Y chromosome [Source:MGI Symbol;Acc:MGI:894810]
	Ddx3y		7.17E-03	DEAD (Asp-Glu-Ala-Asp) box polypeptide 3, Y-linked [Source:MGI Symbol;Acc:MGI:1349406]
	Eif2s3y		2.33E-02	eukaryotic translation initiation factor 2, subunit 3, structural gene Y-linked [Source:MGI Symbol;Acc:MGI:1349430]
	Cd209a		1.29E-03	CD209a antigen [Source:MGI Symbol;Acc:MGI:2157942]
	Slamf8		1.82E-02	SLAM family member 8 [Source:MGI Symbol;Acc:MGI:1921998]
	Kdm5d		6.08E-03	lysine (K)-specific demethylase 5D [Source:MGI Symbol;Acc:MGI:99780]
	Acod1		1.58E-02	aconitate decarboxylase 1 [Source:MGI Symbol;Acc:MGI:103206]
	Cd40		1.01E-03	CD40 antigen [Source:MGI Symbol;Acc:MGI:88336]
	AI427809		9.54E-03	expressed sequence AI427809 [Source:MGI Symbol;Acc:MGI:2140270]
	Esrrg		1.90E-02	estrogen-related receptor gamma [Source:MGI Symbol;Acc:MGI:1347056]
	Sash1	Downregulation	3.39E-02	SAM and SH3 domain containing 1 [Source:MGI Symbol;Acc:MGI:1917347]
	Cspg4		7.67E-04	chondroitin sulfate proteoglycan 4 [Source:MGI Symbol;Acc:MGI:2153093]
	E330009J07Rik		1.06E-04	RIKEN cDNA E330009J07 gene [Source:MGI Symbol;Acc:MGI:2444256]
	Dusp1		3.22E-03	dual specificity phosphatase 1 [Source:MGI Symbol;Acc:MGI:105120]
	Sik1		5.85E-03	salt inducible kinase 1 [Source:MGI Symbol;Acc:MGI:104754]
	E230001N04Rik		3.23E-03	RIKEN cDNA E230001N04 gene [Source:MGI Symbol;Acc:MGI:2443549]
	Igf2bp3		1.06E-04	insulin-like growth factor 2 mRNA binding protein 3 [Source:MGI Symbol;Acc:MGI:1890359]
	Nov		1.18E-02	nephroblastoma overexpressed gene [Source:MGI Symbol;Acc:MGI:109185]
	Smad6		2.54E-04	SMAD family member 6 [Source:MGI Symbol;Acc:MGI:1336883]
	Tln2		1.37E-04	talin 2 [Source:MGI Symbol;Acc:MGI:1917799]
40wks vs 4wks	*Pla2g2d*	Upregulation	7.02E-04	phospholipase A2, group IID [Source:MGI Symbol;Acc:MGI:1341796]
	BC051142		3.92E-08	cDNA sequence BC051142 [Source:MGI Symbol;Acc:MGI:3039565]
	Ccdc7a		6.58E-06	coiled-coil domain containing 7A [Source:MGI Symbol;Acc:MGI:1921953]
	Hmcn1		8.08E-08	hemicentin 1 [Source:MGI Symbol;Acc:MGI:2685047]
	AI427809		1.60E-05	expressed sequence AI427809 [Source:MGI Symbol;Acc:MGI:2140270]
	C130026I21Rik		8.72E-07	RIKEN cDNA C130026I21 gene [Source:MGI Symbol;Acc:MGI:3612702]
	Uty		3.15E-03	ubiquitously transcribed tetratricopeptide repeat gene, Y chromosome [Source:MGI Symbol;Acc:MGI:894810]
	Esrrg		1.04E-04	estrogen-related receptor gamma [Source:MGI Symbol;Acc:MGI:1347056]
	Lipn		7.86E-05	lipase, family member N [Source:MGI Symbol;Acc:MGI:1917416]
	Dpysl3		9.99E-05	dihydropyrimidinase-like 3 [Source:MGI Symbol;Acc:MGI:1349762]
	Zfp979	Downregulation	1.56E-04	zinc finger protein 979 [Source:MGI Symbol;Acc:MGI:2148252]
	Zfp979		1.56E-04	zinc finger protein 979 [Source:MGI Symbol;Acc:MGI:2148252]
	Cd93		9.68E-06	CD93 antigen [Source:MGI Symbol;Acc:MGI:106664]
	Sik1		4.10E-03	salt inducible kinase 1 [Source:MGI Symbol;Acc:MGI:104754]
	Fhdc1		1.63E-02	FH2 domain containing 1 [Source:MGI Symbol;Acc:MGI:2684972]
	Hfm1		1.99E-02	HFM1, ATP-dependent DNA helicase homolog [Source:MGI Symbol;Acc:MGI:3036246]
	Arhgap29		4.29E-05	Rho GTPase activating protein 29 [Source:MGI Symbol;Acc:MGI:2443818]
	Smad6		2.39E-05	SMAD family member 6 [Source:MGI Symbol;Acc:MGI:1336883]
	Egfem1		2.16E-06	EGF-like and EMI domain containing 1 [Source:MGI Symbol;Acc:MGI:1922990]
	Igf2bp3		1.82E-05	insulin-like growth factor 2 mRNA binding protein 3 [Source:MGI Symbol;Acc:MGI:1890359]

Table S4: Up-regulated and downregulated gene sets in KO AMs.

a. GO: Biological process pathways

Comparison (WT	Pathway_ID	Set Size	Mean Log FC	FDR
8wks vs 4wks	GO:0045087 innate immune response	422	4.961	2.09E-03
	GO:0043207 response to external biotic stimulus	469	4.578	3.98E-03
	GO:0051707 response to other organism	469	4.578	3.98E-03
	GO:0098542 defense response to other organism	279	4.514	3.98E-03
	GO:0002252 immune effector process	443	4.485	3.98E-03
	GO:0009607 response to biotic stimulus	495	4.056	2.19E-02
	GO:0019882 antigen processing and presentation	71	4.002	2.28E-02
	GO:0009615 response to virus	187	3.963	2.28E-02
	GO:0050776 regulation of immune response	426	4.078	2.28E-02
	GO:0051607 defense response to virus	154	3.917	2.28E-02
	GO:0034341 response to interferon-gamma	63	3.771	3.62E-02
	GO:0048002 antigen processing and presentation	43	3.766	3.62E-02
	GO:0001819 positive regulation of cytokine produc	262	3.711	4.22E-02
	GO:0002443 leukocyte mediated immunity	208	3.807	4.22E-02
	GO:0001817 regulation of cytokine production	389	3.674	4.22E-02
	GO:0002250 adaptive immune response	227	3.640	4.22E-02
	GO:0009617 response to bacterium	290	3.627	4.22E-02
	GO:0019725 cellular homeostasis	466	3.646	4.22E-02
12wks vs 4wks	GO:0045087 innate immune response	422	4.650	9.27E-03
	GO:0043207 response to external biotic stimulus	469	4.155	1.78E-02
	GO:0051707 response to other organism	469	4.155	1.78E-02
	GO:0098542 defense response to other organism	279	4.168	1.78E-02
	GO:0002252 immune effector process	443	4.146	1.78E-02
	GO:0009607 response to biotic stimulus	495	4.026	2.42E-02

b. KEGG metabolism

Comparison (WT	Pathway_ID	Set Size	Mean Log FC	FDR
8wks vs 4wks	mmu04514 Cell adhesion molecules (CAMs)	60	4.048	1.14E-02
	mmu04612 Antigen processing and presentation	55	3.873	1.14E-02
12wks vs 4wks	mmu04514 Cell adhesion molecules (CAMs)	60	3.795	2.65E-02
	mmu04612 Antigen processing and presentation	55	3.616	2.65E-02
40wks vs 4wks	mmu04514 Cell adhesion molecules (CAMs)	60	4.270	5.94E-03
	mmu04612 Antigen processing and presentation	55	-3.614	1.97E-02

c. KEGG disease

Comparison (WT	Pathway_ID	Set Size	Mean Log FC	FDR
8wks vs 4wks	mmu05416 Viral myocarditis	48	3.45	8.32E-03
	mmu05164 Influenza A	125	3.29	1.25E-02
	mmu05169 Epstein-Barr virus infection	167	3.05	1.25E-02
	mmu05152 Tuberculosis	128	3.15	1.36E-02
	mmu05145 Toxoplasmosis	79	2.83	4.35E-02
	mmu05168 Herpes simplex infection	158	2.45	4.35E-02
	mmu05330 Allograft rejection	27	4.15	7.76E-03
	mmu05322 Systemic lupus erythematosu	52	3.76	8.32E-03
	mmu05150 Staphylococcus aureus infecti	31	3.34	9.39E-03
	mmu05320 Autoimmune thyroid disease	26	3.34	1.03E-02
	mmu05332 Graft-versus-host disease	27	3.21	1.25E-02
	mmu04940 Type I diabetes mellitus	30	2.66	3.32E-02
	mmu05310 Asthma	11	2.48	4.35E-02
12wks vs 4wks	mmu05416 Viral myocarditis	48	3.38	1.59E-02
	mmu05150 Staphylococcus aureus infecti	31	3.37	1.59E-02
	mmu05164 Influenza A	125	2.92	2.28E-02
	mmu05169 Epstein-Barr virus infection	167	2.88	2.28E-02
	mmu05152 Tuberculosis	128	2.76	2.60E-02
	mmu05145 Toxoplasmosis	79	2.49	4.39E-02
	mmu05330 Allograft rejection	27	3.74	1.59E-02
	mmu05322 Systemic lupus erythematosu	52	3.28	1.59E-02
	mmu05320 Autoimmune thyroid disease	26	3.12	2.28E-02
	mmu05332 Graft-versus-host disease	27	2.95	2.54E-02
	mmu04940 Type I diabetes mellitus	30	2.83	2.71E-02
	mmu05310 Asthma	11	2.99	3.80E-02
40wks vs 4wks	mmu05416 Viral myocarditis	48	4.11	5.29E-03
	mmu05150 Staphylococcus aureus infecti	31	3.74	6.14E-03
	mmu05164 Influenza A	125	3.40	7.13E-03
	mmu05169 Epstein-Barr virus infection	167	3.49	7.13E-03
	mmu05330 Allograft rejection	27	4.67	1.97E-03
	mmu05322 Systemic lupus erythematosu	52	3.81	6.14E-03
	mmu05320 Autoimmune thyroid disease	26	3.49	6.70E-03
	mmu05332 Graft-versus-host disease	27	3.93	9.98E-03

Table S5: Ten most significantly upregulated and downregulated genes in WT and KO AMs.

Comparison (KO vs WT)	Gene name	Gene expression	FDR	Gene description
4wks	*Nuak2*	Upregulation	2.91E-02	NUAK family, SNF1-like kinase, 2 [Source:MGI Symbol;Acc:MGI:1921387]
	Arrdc3		2.91E-02	Arrestin domain containing 3 [Source:MGI Symbol;Acc:MGI:2145242]
	Myc	Downregulation	3.39E-02	Myelocytomatosis oncogene [Source:MGI Symbol;Acc:MGI:97250]
	Kl		3.39E-02	Klotho [Source:MGI Symbol;Acc:MGI:1101771]
8wks	*Herpud1*	Upregulation	1.84E-02	Homocysteine-inducible, endoplasmic reticulum stress-inducible, ubiquitin-like domain member 1 [Source:MGI Symbol;Acc:MGI:1927406]
	Snx27		2.31E-02	Sorting nexin family member 27 [Source:MGI Symbol;Acc:MGI:1923992]
	Galnt7		2.42E-02	Polypeptide N-acetylgalactosaminyltransferase 7 [Source:MGI Symbol;Acc:MGI:1349449]
	Cenpf		2.91E-02	Centromere protein F [Source:MGI Symbol;Acc:MGI:1313302]
	Cenpe		2.91E-02	Centromere protein E [Source:MGI Symbol;Acc:MGI:1098230]
	Crip1		2.91E-02	Cysteine-rich protein 1 (intestinal) [Source:MGI Symbol;Acc:MGI:88501]
	Dnajb9		2.91E-02	DnaJ heat shock protein family (Hsp40) member B9 [Source:MGI Symbol;Acc:MGI:1351618]
	Runx1		2.91E-02	Runt related transcription factor 1 [Source:MGI Symbol;Acc:MGI:99852]
	Fndc3a		3.19E-02	Fibronectin type III domain containing 3A [Source:MGI Symbol;Acc:MGI:1196463]
	Cytip		3.48E-02	Cytohesin 1 interacting protein [Source:MGI Symbol;Acc:MGI:2183535]
	Pilra	Downregulation	7.72E-03	Paired immunoglobin-like type 2 receptor alpha [Source:MGI Symbol;Acc:MGI:2450529]
	Hdac4		2.31E-02	Histone deacetylase 4 [Source:MGI Symbol;Acc:MGI:3036234]
	Rnase6		2.31E-02	Ribonuclease, RNase A family, 6 [Source:MGI Symbol;Acc:MGI:1925666]
	Btd		2.31E-02	Biotinidase [Source:MGI Symbol;Acc:MGI:1347001]
	Pilrb2		2.31E-02	Paired immunoglobin-like type 2 receptor beta 2 [Source:MGI Symbol;Acc:MGI:2450535]
	Mvk		2.31E-02	Mevalonate kinase [Source:MGI Symbol;Acc:MGI:107624]
	Arpin		2.91E-02	Actin-related protein 2/3 complex inhibitor [Source:MGI Symbol;Acc:MGI:1917670]
	Slc2a6		2.91E-02	Solute carrier family 2 (facilitated glucose transporter), member 6 [Source:MGI Symbol;Acc:MGI:2443286]
	Mcub		3.19E-02	mitochondrial calcium uniporter dominant negative beta subunit [Source:MGI Symbol;Acc:MGI:1914065]
	Paqr7		3.19E-02	Progestin and adipoQ receptor family member VII [Source:MGI Symbol;Acc:MGI:1919154]
12wks	*Tmem216*	Upregulation	2.63E-06	Transmembrane protein 216 [Source:MGI Symbol;Acc:MGI:1920020]
	Ddx46		4.54E-06	DEAD (Asp-Glu-Ala-Asp) box polypeptide 46 [Source:MGI Symbol;Acc:MGI:1920895]
	Acaa1b		1.38E-05	Acetyl-Coenzyme A acyltransferase 1B [Source:MGI Symbol;Acc:MGI:3605455]
	Gmpr		2.79E-05	Guanosine monophosphate reductase [Source:MGI Symbol;Acc:MGI:1913605]
	Tc2n		3.12E-05	Tandem C2 domains, nuclear [Source:MGI Symbol;Acc:MGI:1921663]
	Bzw2		3.73E-05	Basic leucine zipper and W2 domains 2 [Source:MGI Symbol;Acc:MGI:1914162]
	Dennd4a		5.04E-05	DENN/MADD domain containing 4A [Source:MGI Symbol;Acc:MGI:2142979]
	Pparg		5.05E-05	Peroxisome proliferator activated receptor gamma [Source:MGI Symbol;Acc:MGI:97747]
	Prom1		6.97E-05	Prominin 1 [Source:MGI Symbol;Acc:MGI:1100886]
	Fabp1		9.26E-05	Fatty acid binding protein 1, liver [Source:MGI Symbol;Acc:MGI:95479]
	Slc3a2	Downregulation	5.47E-07	Solute carrier family 3 (activators of dibasic and neutral amino acid transport), member 2 [Source:MGI Symbol;Acc:MGI:96955]
	Sc5d		5.47E-07	Sterol-C5-desaturase [Source:MGI Symbol;Acc:MGI:1353611]
	Fdft1		5.47E-07	Farnesyl diphosphate farnesyl transferase 1 [Source:MGI Symbol;Acc:MGI:102706]
	Fdps		5.47E-07	Farnesyl diphosphate synthetase [Source:MGI Symbol;Acc:MGI:104888]
	Ninj1		5.47E-07	Ninjurin 1 [Source:MGI Symbol;Acc:MGI:1196617]
	Slc2a6		5.47E-07	Solute carrier family 2 (facilitated glucose transporter), member 6 [Source:MGI Symbol;Acc:MGI:2443286]
	Zwint		6.40E-07	ZW10 interactor [Source:MGI Symbol;Acc:MGI:1289227]
	Acss2		6.40E-07	Acyl-CoA synthetase short-chain family member 2 [Source:MGI Symbol;Acc:MGI:1890410]
	Nampt		7.11E-07	Nicotinamide phosphoribosyltransferase [Source:MGI Symbol;Acc:MGI:1929865]
	Msmo1		7.66E-07	Methylsterol monoxygenase 1 [Source:MGI Symbol;Acc:MGI:1913484]
40wks	*H2-Oa*	Upregulation	1.33E-06	Histocompatibility 2, O region alpha locus [Source:MGI Symbol;Acc:MGI:95924]
	Cd209b		1.33E-06	CD209b antigen [Source:MGI Symbol;Acc:MGI:1916415]
	Slc1a3		8.93E-06	Solute carrier family 1 (glial high affinity glutamate transporter), member 3 [Source:MGI Symbol;Acc:MGI:99917]
	Hmcn1		3.40E-05	Hemicentin 1 [Source:MGI Symbol;Acc:MGI:2685047]
	H2-DMb1		3.40E-05	Histocompatibility 2, class II, locus Mb1 [Source:MGI Symbol;Acc:MGI:95922]
	Klhl41		1.71E-04	Kelch-like 41 [Source:MGI Symbol;Acc:MGI:2683854]
	4930502E18Rik		1.71E-04	RIKEN cDNA 4930502E18 gene [Source:MGI Symbol;Acc:MGI:1922263]
	Zfhx3		1.71E-04	Zinc finger homeobox 3 [Source:MGI Symbol;Acc:MGI:99948]
	H2-Eb1		1.85E-04	Histocompatibility 2, class II antigen E beta [Source:MGI Symbol;Acc:MGI:95901]
	Ciita		2.04E-04	Class II transactivator [Source:MGI Symbol;Acc:MGI:108445]
	Fpr1	Downregulation	1.60E-04	Formyl peptide receptor 1 [Source:MGI Symbol;Acc:MGI:107443]
	Emilin1		2.84E-04	Elastin microfibril interfacer 1 [Source:MGI Symbol;Acc:MGI:1926189]
	Fn1		3.40E-04	Fibronectin 1 [Source:MGI Symbol;Acc:MGI:95566]
	Egfem1		3.40E-04	EGF-like and EMI domain containing 1 [Source:MGI Symbol;Acc:MGI:1922990]
	Ubl3		6.48E-04	Ubiquitin-like 3 [Source:MGI Symbol;Acc:MGI:1344373]
	Clec4e		8.29E-04	C-type lectin domain family 4, member e [Source:MGI Symbol;Acc:MGI:1861232]
	Tspan32		8.82E-04	Tetraspanin 32 [Source:MGI Symbol;Acc:MGI:1350360]
	Inka1		1.19E-03	Inka box actin regulator 1 [Source:MGI Symbol;Acc:MGI:1915426]
	Ptgr1		1.27E-03	Prostaglandin reductase 1 [Source:MGI Symbol;Acc:MGI:1914353]
	Psmd11		1.63E-03	Proteasome (prosome, macropain) 26S subunit, non-ATPase, 11 [Source:MGI Symbol;Acc:MGI:1916327]

Table S6: Upregulated and downregulated gene sets in WT and KO AMs.

GO: Molecular Function

Comparison (KO vs WT	Pathway_ID	Set Size	Mean Log FC	FDR
4wks	GO:0003735 structural constituent of ribosome	143	-4.909	8.52E-04
8wks	GO:0030545 receptor regulator activity	123	-3.762	2.52E-02
	GO:0042277 peptide binding	153	-3.936	1.69E-02
	GO:0008009 chemokine activity	19	-3.935	1.69E-02
	GO:0003735 structural constituent of ribosome	143	-4.198	1.43E-02

KEGG disease

Comparison (KO vs WT	Pathway_ID	Set Size	Mean Log FC	FDR
12wks	mmu05012 Parkinson's disease	117	2.581	2.78E-02
	mmu05016 Huntington's disease	152	3.886	2.47E-03
	mmu04932 Non-alcoholic fatty liver disease (NAFLD)	127	2.462	3.60E-02
	mmu05418 Fluid shear stress and atherosclerosis	94	-2.968	1.87E-02
	mmu04940 Type I diabetes mellitus	30	-2.964	1.87E-02
	mmu05320 Autoimmune thyroid disease	26	-2.751	2.45E-02
	mmu05332 Graft-versus-host disease	27	-2.872	1.95E-02
	mmu05330 Allograft rejection	27	-2.989	1.87E-02
	mmu05134 Legionellosis	51	-2.943	1.87E-02
	mmu05167 Kaposi's sarcoma-associated herpesvirus infec	149	-3.534	5.95E-03
	mmu05162 Measles	93	-2.832	1.94E-02
	mmu05169 Epstein-Barr virus infection	167	-4.442	4.53E-04
	mmu05160 Hepatitis C	116	-2.757	1.95E-02
	mmu05168 Herpes simplex infection	158	-3.156	1.30E-02
	mmu05164 Influenza A	125	-3.305	1.04E-02
40wks	mmu05016 Huntington's disease	152	3.046	4.52E-02
	mmu04940 Type I diabetes mellitus	30	2.661	4.70E-02
	mmu05330 Allograft rejection	27	3.027	4.60E-02
	mmu05134 Legionellosis	51	2.723	4.60E-02
	mmu05167 Kaposi's sarcoma-associated herpesvirus infec	149	3.065	4.52E-02
	mmu05168 Herpes simplex infection	158	2.819	4.60E-02
	mmu05164 Influenza A	125	3.000	4.52E-02
	mmu05418 Fluid shear stress and atherosclerosis	94	-2.688	4.60E-02
	mmu05169 Epstein-Barr virus infection	167	-3.417	2.77E-02

GO: Biological process

Comparison (KO vs WT	Pathway_ID	Set Size	Mean Log FC	FDR
12wks	GO:0045087 innate immune response	422	-5.641	5.59E-05
	GO:0009607 response to biotic stimulus	495	-5.333	8.08E-05
	GO:0043207 response to external biotic stimulus	469	-5.316	8.08E-05
	GO:0051707 response to other organism	469	-5.316	8.08E-05
	GO:0098542 defense response to other organisn	279	-5.241	1.13E-04
	GO:0034097 response to cytokine	465	-4.614	1.74E-03
	GO:0001817 regulation of cytokine production	389	-4.592	1.74E-03
	GO:0009617 response to bacterium	290	-4.466	2.75E-03
	GO:0001816 cytokine production	430	-4.427	2.75E-03
	GO:0002252 immune effector process	443	-4.410	2.75E-03
	GO:0006954 inflammatory response	348	-4.206	6.53E-03
	GO:0071345 cellular response to cytokine stimulu	393	-4.077	9.89E-03
	GO:0051607 defense response to virus	154	-3.987	1.43E-02
	GO:0009615 response to virus	187	-3.893	1.86E-02
	GO:0034341 response to interferon-gamma	63	-3.843	3.14E-02
	GO:0042742 defense response to bacterium	111	-3.749	3.18E-02
	GO:0001819 positive regulation of cytokine produ	262	-3.709	3.18E-02
	GO:0006412 translation	493	4.041	1.05E-02

KEGG Metabolism

Comparison (KO vs WT	Pathway_ID	Set Size	Mean Log FC	FDR
4wks	mmu03010 Ribosome	133	-5.421	2.18E-05
8wks	mmu04621 NOD-like receptor signaling pathway	133	-3.300	4.48E-02
	mmu00100 Steroid biosynthesis	15	-3.432	4.48E-02
	mmu03010 Ribosome	133	-4.142	3.21E-02
12wks	mmu00190 Oxidative phosphorylation	112	3.124	2.88E-02
	mmu03010 Ribosome	133	5.576	1.05E-05
	mmu04978 Mineral absorption	25	-3.301	2.88E-02
	mmu03050 Proteasome	44	-3.211	2.88E-02
	mmu04612 Antigen processing and presentation	55	-3.401	2.14E-02
	mmu04668 TNF signaling pathway	90	-3.368	2.14E-02
	mmu04621 NOD-like receptor signaling pathway	133	-4.040	2.52E-03
	mmu00100 Steroid biosynthesis	15	-5.414	5.18E-04
40wks	mmu03010 Ribosome	133	-4.199	3.74E-03
